# The Interactions of Parasite Calreticulin With Initial Complement Components: Consequences in Immunity and Virulence

**DOI:** 10.3389/fimmu.2020.01561

**Published:** 2020-07-23

**Authors:** Galia Ramírez-Toloza, Lorena Aguilar-Guzmán, Carolina Valck, Viviana P. Ferreira, Arturo Ferreira

**Affiliations:** ^1^Department of Preventive Veterinary Medicine, Faculty of Veterinary Medicine and Livestock Sciences, University of Chile, Santiago, Chile; ^2^Department of Pathology, Faculty of Veterinary Medicine and Livestock Sciences, University of Chile, Santiago, Chile; ^3^Department of Immunology, ICBM, Faculty of Medicine, University of Chile, Santiago, Chile; ^4^Department of Medical Microbiology and Immunology, College of Medicine and Life Sciences, University of Toledo, Toledo, OH, United States

**Keywords:** *Trypanosoma cruzi*, complement, calreticulin, C1q, host-parasite interaction, host immune evasion

## Abstract

Because of its capacity to increase a physiologic inflammatory response, to stimulate phagocytosis, to promote cell lysis and to enhance pathogen immunogenicity, the complement system is a crucial component of both the innate and adaptive immune responses. However, many infectious agents resist the activation of this system by expressing or secreting proteins with a role as complement regulatory, mainly inhibitory, proteins. *Trypanosoma cruzi*, the causal agent of Chagas disease, a reemerging microbial ailment, possesses several virulence factors with capacity to inhibit complement at different stages of activation. *T. cruzi* calreticulin (TcCalr) is a highly-conserved, endoplasmic reticulum-resident chaperone that the parasite translocates to the extracellular environment, where it exerts a variety of functions. Among these functions, TcCalr binds C1, MBL and ficolins, thus inhibiting the classical and lectin pathways of complement at their earliest stages of activation. Moreover, the TcCalr/C1 interaction also mediates infectivity by mimicking a strategy used by apoptotic cells for their removal. More recently, it has been determined that these Calr strategies are also used by a variety of other parasites. In addition, as reviewed elsewhere, TcCalr inhibits angiogenesis, promotes wound healing and reduces tumor growth. Complement C1 is also involved in some of these properties. Knowledge on the role of virulence factors, such as TcCalr, and their interactions with complement components in host–parasite interactions, may lead toward the description of new anti-parasite therapies and prophylaxis.

## Introduction

The complement system (C), essential in both the innate and adaptive immune responses, increases physiologic inflammation, stimulates microbial phagocytosis and their lysis, and promotes the elimination of a large variety of aggressive microorganisms by enhancing their immunogenicity. Some activated C components and derived molecules, opsonize a variety of microorganisms and apoptotic cells promoting their phagocytosis and destruction inside the phagocyte (1). However, pathogens such as viruses, bacteria, fungi and parasites, utilize some surface proteins and receptors to evade C during its activation (1).

*Trypanosoma cruzi* calreticulin (TcCalr), similar to calreticulin from other species, including human (HuCALR), is a multifunctional endoplasmic reticulum-resident chaperone, that the parasite translocates to the extracellular environment, where TcCalr participates in C evasion and infection, with important consequences in virulence. Thus, TcCalr is a *bona fide* virulence factor. Calr from other important parasite species shares several of these properties with TcCalr. These issues are reviewed herein. TcCalr also participates in the control of angiogenesis and tumor growth, as reviewed elsewhere (2).

## Complement Activation and Regulation: a Brief Overview

C consists of soluble and membrane-bound molecules that are activated through a stringently regulated proteolytic cascade (3). Activation may occur through the classical (CP), alternative (AP), and lectin (LP) pathways. The CP is initiated by the recognition, by C1, of antibodies aggregated on foreign antigens, or by acute phase proteins identifying danger signals on a microbial aggressor. The LP is activated by mannose-binding lectin (MBL) or by ficolins recognizing a variety of bacterial motifs. Conversely, spontaneous hydrolysis of C3, near cell surfaces, produces a constitutive AP activation, which is tightly controlled by C regulatory proteins present on host cells (Factor I, C4-binding protein, decay-accelerating factor, membrane co-factor protein, C receptor 1) or in plasma (Factor H, C1-inhibitor, S-protein, clusterin, CD59). These proteins limit amplification of the downstream cascade (3, 4).

C activation generates split products with opsonizing, pro-inflammatory and immune-stimulating properties (3). The three activation pathways converge in the generation of C3 convertases that continuously cleave C3 into C3a and C3b, as well as C5 convertases that produce the split products C5a and C5b. C5b, in conjunction with C6–C9, form the membrane attack complex (MAC) and lyse the pathogen (3).

## C1 and Calreticulin Interaction Promotes Phagocytosis

In mammals, C1 is a highly complex protein, composed by eighteen polypeptide chains, grouped in six heterotrimeric units, each carrying the products of 3 genes, A, B, C. Each trimer has several functional sites located on both a collagen-like (cC1q) and a globular head (gC1q) regions (5). Each globular head (ghA, ghB, and ghC) has special affinity for the CH2 and CH3 domains of IgG and IgM molecules, respectively, or for other unrelated molecules (5).

Beyond its role as a pattern recognition receptor (PRR), C1 binds to a wide variety of phagocytic cells, resulting in the induction of cell-specific responses such as phagocytosis, cellular activation, release of biological mediators and expression of adhesion molecules, promoting inflammation (6). At least four C1q binding cell surface receptors have been identified: CR1 (CD35), receptor for C3b; C1q-Rp (CD93), a 120 kDa O-sialoglycoprotein; gC1q-R/p33, a 33kDa homotrimeric protein, and cC1q-R/CR, a 60 kDa protein (5, 6). The 33 kDa molecule has high affinity for the globular heads while the 60 kDa molecule, also known as collectin receptor, binds to the collagenous tails and its N-terminal sequence is 100% identical with Calr (5).

Calr is a 46 kDa multifunctional protein, mainly located in the endoplasmic reticulum (ER) and highly conserved in all species, including plants and microorganisms (2, 7, 8). Calr is involved in Ca^2+^ homeostasis and in other important functions inside and outside the cell, including: cardiogenesis, adipocyte differentiation, cellular stress responses, wound healing and immunity (9). Its structure comprises three main domains: N-terminal globular, flexible proline-rich P intermediate arm-like and C-carboxyl terminal (7, 9).

Both, C1q and MBL bind to apoptotic cells and stimulate phagocytosis by ligation of Calr on the phagocyte surface, which binds to the endocytic receptor protein CD91 (10). Phagocytic cells, monocyte-derived macrophages and dendritic cells express and secrete Calr as a C1q receptor. On the cell, Calr bridges the phagocytic cell and the target (apoptotic cell or an immune complex), promoting removal (6). Activated macrophages secrete Calr, which binds to the surface of viable target cells and marks them for removal by programmed cell phagocytosis (11). Additionally, Calr is found on the surface of apoptotic cells acting as a damage-associated molecular pattern (DAMP), responsible for the immunogenicity of apoptotic cancerous cells (12–14). Binding of C1q to cell-bound Calr results in opsonization (15). Most important, the pro-phagocytic Calr/C1q/C1qR interaction is used by different parasites to promote infectivity.

## *Trypanosoma Cruzi* Evades the Complement System: the Role of Calreticulin

Chagas disease, is a zoonotic and chronic parasitic illness affecting 7–8 million people worldwide, that may be symptomatic in about 30% of those infected, leading to incapacitating situations in some of them. The disease is currently endemic in 21 Latin-American countries and, due to migration of chronically infected individuals, is now a global concern (16). Its causal agent, the flagellated protozoan *T. cruzi*, is an obligatory intracellular infectious agent transmitted by triatomine vectors, but also by congenital route, blood transfusions, organ transplantation or by ingesting contaminated food and beverages (17). In all these routes of infection, once trypomastigotes (infective form in mammalian host) reach the bloodstream, the parasite, using different proteins and mechanisms, bypasses C-mediated lysis (18), and disseminates to many tissues during the acute phase (19). There are a variety of molecules involved in C immune evasion in *T. cruzi*. Among them, TcCalr plays an important role.

Bloodstream trypomastigotes, amastigotes (intracellular replicative stage in host cells) and metacyclic trypomastigotes (infective forms present in vector dejections) are resistant to the C-mediated lysis (20). Instead, epimastigotes, the replicative and non-infective form of the parasite, are highly susceptible (21–23). The ability to resist C differs among the parasite developmental stage (24) and strains (21).

Several molecules present on the parasite have been identified as resistance mediators, at different levels of the C cascade. Moreover, trypomastigotes capture inhibitory host components, which are used to inhibit the C activation on the parasite surface, such as: plasma-membrane derived-vesicles (PMV) (25, 26), *T. cruzi* trypomastigotes-decay accelerating factor (T-DAF) (27, 28), *T. cruzi* C regulatory protein (CRP) (29–32); Factor H (FH) (33), gp58/68 (34), and C2 receptor inhibitor trispanning (CRIT) (21, 35) (Table 1). The molecular inhibitory mechanisms of these proteins are only partially known. Some of these molecules play a central role in the inhibition of C3 and/or C5 convertases. The inhibition of these key enzymes may have important biological consequences, such as: (i) inhibition of C-mediated lysis, (ii) a decrease in the C3a and C5a (anaphylotoxins) generation (these small C fragments are essential in the recruitment of blood cells to the infection site), and (iii) a decreased opsonization, which mediates phagocytosis of pathogens during infection (25).

**Table 1:** Regulatory proteins playing a role in *Trypanosoma cruzi* complement system immune evasion.

**Complement regulatory protein**	**Specific functions**	**Complement pathway affected**	**References**
**COMPLEMENT REGULATORY PROTEINS PRESENT ON THE** ***T. CRUZI*** **SURFACE**			
*Trypanosoma cruzi* calreticulin (TcCalr)	TcCalr is a 45 kDa protein that binds to C1 (C1q, C1r, and C1s), and also binds to MBL and ficolins (L-Ficolin).	CP and AP	(36–38)
Trypomastigote Decay-Accelerating Factor (T-DAF)	T-DAF is a 87–93 kDa glycoprotein that interferes with assembly of the C3 and C5 convertase of both CP and AP.	CP, LP (probably) and AP	(27, 28)
*Trypanosoma cruzi* Complement C2 Receptor Inhibitor Trispanning Protein (CRIT)	CRIT is a 32 kDa protein that inhibits the C2 cleavage by C1s and MASP2 and impairs C3 convertase formation.	CP and LP	(21, 35)
*Trypanosoma cruzi* Complement Regulatory Protein (TcCRP)	TcCRP is a surface-anchored glycoprotein also named gp160 that binds C3b and C4b, inhibiting the CP and AP C3 convertase.	CP, LP (probably) and AP	(29–32)
Glycoprotein 58/68 (Gp58/68)	GP58/68 is a 58-68 kDa protein that inhibits the C3 convertase formation by binding factor B.	AP	(34)
**COMPLEMENT REGULATORY PROTEIN FROM THE HOST USED BY** ***T. CRUZI***			
Factor H (FH)	FH binds to trypomastigotes covered by sialic acid probably accelerating the decay of C3 convertase.	AP	(33)
**OTHER PROTEINS WITH COMPLEMENT REGULATORY FUNCTIONS IN** ***T. CRUZI***			
*T. cruzi* induced membrane-derived vesicles from host cells or microvesicles (MV)	MVs from different types of cells interact with C3 convertase	CP and LP	(25, 26)

*CP, Classical pathway; LP, Lectin pathway; AP, Alternative pathway; C, Complement system*.

**Figure 1:**
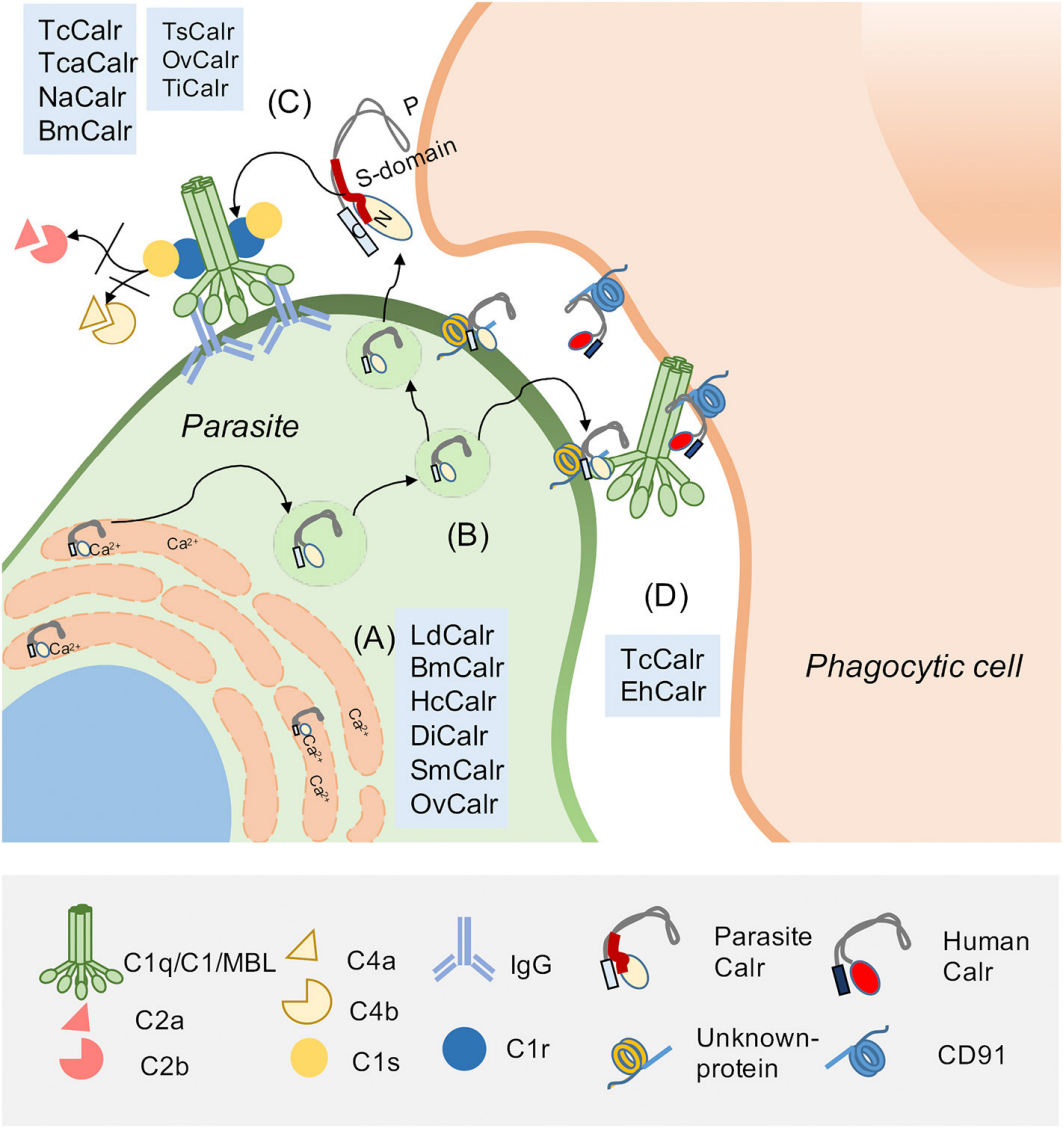
Parasite Calreticulin participates in complement (C) evasion and in infectivity. Calreticulin (Calr), highly conserved and pleiotropic, is mainly found in the endoplasmic reticulum (ER), but also in the extracellular environment. (A) In the ER, Calr is a chaperone and Ca^2+^ binding protein. These properties are also described for Calrs from other parasites, such as: *L. donovani* (LdCalr), *B. malayi* (BmCalr), *H. contortus* (HcCalr), *D. immitis* (DiCalr), *S. mansoni* (SmCalr) and *O. viverrini (OvCalr)*. (B) Extracellularly, the Calr S-domain (a fragment located between N and P domains) interacts with C system molecules such as (C) C1q, C1 complex and MBL, inhibiting C activation. This property has been described for human Calr (HuCALR) and parasitic Calrs such as *T*. *cruzi calreticulin* (TcCalr), *T. carassii* (TcaCalr), *N. americanum* (NaCalr), BmCalr, *T. spiralis* (TsCalr), OvCalr, and *T. infestans* (TiCalr). Additionally, the C1/Calr interaction on the parasite is used by *T. cruzi* and *E. histolytica* to promote infectivity. (D) TcCalr and *E. histolytica* Calr (EhCalr) on the parasite surface bind C1q. This interaction is recognized by HuCALR present on phagocytes as an “eat me” signal. This Calr/C1q/HuCALR interaction, that promotes phagocytosis, was previously described as a mechanism to promote the clearance of apoptotic cells, which overexpress Calr as a damage-associated molecular pattern (DAMP) on their surface. This Calr on apoptotic cells is recognized by the pattern-recognition receptor (PRR) C1q, which is recognized by a C1q receptor on the phagocytic cells, identified as HuCALR. Calr, which does not have a transmembrane tail, binds to CD91 on the phagocyte.

TcCalr, similar to its human counterpart, resides in the ER, where it modulates Ca^2+^ homeostasis and participates as a chaperone protein (39). However, TcCalr is also located in the Golgi, reservosomes, flagellar pocket, cell surface, cytosol, nucleus and kinetoplast. Large quantities of TcCalr accumulate in the kinetoplast, apparently as a previous step to its translocation to the parasite exterior (36, 39, 40). This parasitic protein shares 50% of homology with HuCALR (41) and with its three domains (42): N, P, and C. Within the N and P domains, TcCalr has an S-domain (aa 159–281) that specifically interacts with C1 (43, 44) (Figure 1).

The TcCalr/C1 interaction promotes important functions in the host-parasite interplay. TcCalr competes with the (C1r-C1s)_2_ tetrameric complex for binding to the collagenous tails of C1q, interfering with the C1s-mediated cleavage of C4 and C2 and thus CP activation (36). TcCalr binds both serine-proteases, C1r and C1s, but only TcCalr-C1r binding inhibits the C4-activating function (37). Additionally, TcCalr competes with the serine proteases, but does not displace them from preformed C1 (37). This fact may be explained by the strong interaction between the enzymatic tetramer (C1r_2_, C1s_2_) and C1q (45). TcCalr inhibits C more efficiently than HuCALR and these functional differences may be explained, at least in part, by comparative crystallographic studies that have identified conformational rearrangements in TcCalr and HuCALR (46) and some aminoacidic substitutions that confer differences in polarity and spatial stability (47). TcCalr not only inhibits the CP; it also binds MBL and Ficolins, inhibiting the LP (38). L-, but not H-Ficolin binds to TcCalr, but this binding does not interfere with lipoteichoic acid binding to L-Ficolin and its activation. Moreover, L-Ficolin binds preferentially to trypomastigotes, rather than to epimastigotes, which translocate significantly lower amounts of TcCalr to their surfaces (38). All these facts have been corroborated *in vivo* by using genetically modified parasites carrying a monoallelic deletion of the *TcCRT* gene or a transgenic version, expressing an extra copy of the gene. The parasites expressing less TcCalr are significantly more susceptible to C-mediated lysis and those overexpressing TcCRT are significantly more resistant to both CP and LP-mediated lysis (48, 49).

## TcCALR/C1 Interaction: Role in Promoting Infectivity

Another important role of the TcCalr/C1 interaction is to promote infectivity. TcCalr is expressed mainly on the trypomastigote flagellum emergence area, where it recruits C1q/C1 (36). This interaction correlates with a TcCalr mRNA level increase in the early infection steps (50). This is corroborated since anti-TcCalr F(ab')_2_ antibody fragments (unable to bind C1 because they lack their Fc domains) inhibit the TcCalr/C1 interaction, thus decreasing infectivity *in vitro* and *in vivo* (50). Non-infective epimastigotes express less TcCalr on their surface (36, 51), which may contribute to their high sensitivity to C and lack of infectivity. In agreement with this notion, when exogenous TcCalr is added, epimastigotes are internalized by fibroblasts, in a C1q-dependent manner (52).

As mentioned, the capacity of the TcCalr/C1q interaction to mediate phagocytosis was originally described for apoptotic cells. C1q and MBL bind to these cells, exposing HuCALR, and stimulating their ingestion by ligation on the phagocyte surface in a HuCALR/C1q-mediated manner (10). We have proposed that the TcCalr/C1q complex is recognized as an “*eat me”* signal on the parasite by host Calr on phagocytes and other cellular types, thus promoting infectivity (Figure 1). In agreement with these findings, Calr-deficient fibroblasts are unable to internalize these parasites (52). Moreover, in mice inoculated with trypomastigotes, carrying a monoallelic *TcCalr* deletion, no parasitemia, nor anti-*T. cruzi* IgG levels are detected, demonstrating that these mutants have a potent restriction in their capacity to infect host cells, due to insufficient Calr expression and consequent reduced resistance to C (49).

Calr is also important in infectivity, as determined in an *ex vivo* model using human placenta explants, which express high HuCALR levels (53–55). In these explants, the TcCalr/C1q/HuCALR synapsis mediates the first stages of *T. cruzi* infection (56). This fact is particularly relevant due to the current high impact of congenital Chagas disease transmission.

TcCalr also binds MBL and Ficolins (36, 37), but the role of TcCalr/MBL or TcCalr/Ficolins interactions in *T. cruzi* infectivity processes has not yet been demonstrated. However, in C-resistant *T. cruzi* strains, MBL seems to participate in the infectivity process while the parasite deactivates the LP (57). However, the ligand for MBL on the parasite surface remains unknown.

## Calreticulin in Other Parasitic Infections

Several functions are shared and conserved, to differing extents, by Calr from different species (41, 58). Calr, is a surprisingly pleiotropic protein, present in all nucleated cells in different organisms including parasites, where it was first described in *Schistosoma mansoni* (59, 60), *Dirofilaria immitis* (61) and *Necator americanus* (62). More recently, Calr has been characterized in *Entamoeba histolytica* (63, 64), *Leishmania donovani* (65), *Trypanosoma carassii* (66), and in the helminths *Brugia malayi* (67), *Haemonchus contortus* (68), *Opisthorchis viverrini* (69, 70), and *Trichinella spiralis* (71).

Structurally, Calr from different species possesses a broad spectrum of sequence conservations and differences (46). *L. donovani* Calr (LdCalr) binds Ca^2+^and RNA sequences (65) and its P-domain is implicated in ER chaperone functions, since a modulation of its expression affects the targeting of proteins associated with virulence, during their trafficking through the parasite secretory pathway (72). Additionally, proteomic approaches indicate LdCalr is an immunostimulatory protein (73, 74). However, its specific role in host-parasite interactions is still unknown.

In *E. histolytica*, a protozoan parasite that causes amebiasis, Calr (EhCalr) participates in several roles related to the host-immune modulation. Quantitative proteomic analysis and an *ex vivo* modeling indicates that EhCalr is an abundant membrane protein expressed in virulent variants (75, 76). EhCalr, from pathogenic and non-pathogenic species, binds C1 and inhibits the CP activation (63). Additionally, EhCalr is exported from the ER to the phagolysosome, where it favors phagocytosis in a C1q-dependent manner (64, 77). EhCalr also interferes in the pathogenesis and host immune response modulation. Thus, *in vitro*, EhCalr acts as an immunogen for the specific activation of peripheral blood mononuclear cells, inducing a Th2 cytokine profile, during the acute phase, and a Th1 profile in the resolution phase (78).

*T. carassii*, a flagellated bloodstream parasite of cyprinid fish, produces anemia during peak parasitemias and it is highly resistant to C-mediated lysis (79, 80). Its Calr (TcaCalr) is a surface protein that binds C1 and inhibits the CP activation (66), but its role in infectivity has not yet been elucidated. *Trypanosoma congolense* Calr (TcoCalr) is an immunogen in mice, delaying parasitemia and increasing survival in challenged animals (81).

In nematodes Calr is also important in immune evasion (2). *Necator americanus* Calr (NaCalr) was first described as a hookworm allergen in infected patients (62). NaCalr does not bind Ca^2+^, but interacts with C1 and inhibits the CP (82). Calr from *B. malayi* (BmCalr), a parasite causing lymphatic filariasis, binds Ca^2+^ and Zinc (67) and interacts with host C1, inhibiting the CP (83). *Haemonchus contortus* is a gastrointestinal parasite of small ruminants that feeds on blood. The N-domain of HcCalr mediates Ca^2+^ binding and blood clotting inhibition. It also binds C1 (68) and C-reactive protein (84), thus inhibiting the CP. The C1 binding sites reside in two sequences present in its N-domain (85). *Trichinella spiralis* activates C in infective larvae, adults and newborn larvae. However, C is primarily activated by the AP (86) and none of these stages bind C1 (87). *T. spiralis* expresses two proteins that bind C1 and inhibit the CP: paramyosin (88) and Calr (TsCalr) (71). Additionally, TsCalr/C1q binding inhibits the C1-induced non-C activation of macrophages (71). In the nematode *Dirofilaria immitis*, a Ca^2+^ binding protein, similar to Calr, was isolated and shown to be immunogenic in chronically-infected microfilaremic dogs (61).

Calrs from the trematodes *Schistosoma mansoni* (SmCalr) and *Schistosoma japonicum* (SjCalr) have been characterized (59, 60, 89, 90). SmCalr is a Ca^2+^ binding protein, mainly present in miracidia and genital organs (59), that participates as T and B cell antigen (60). Both C1 (91) and MBL (92) bind to *S. mansoni*, but the role of SmCalr in this binding or C evasion is unknown. SjCalr participates as an immunomodulatory protein, activating dendritic cells and inducing a Th1 immune response (89). Calr from *Opisthorchis viverrini* (OvCalr), a trematode parasite affecting humans, with carcinogenic effects, is mainly expressed in the reproductive system and its C-domain binds Ca^2+^ (70). OvCalr also binds C1 and inhibits the CP and, additionally, OvCalr is released from the parasite, interfering with cell proliferation, cell migration and sprouting, and stimulates specific antibody production (69).

Hematophagous arthropods also use these Calr-mediated mechanisms to evade C. Thus, Calr from *Triatoma infestans* (TiCalr), the principal vector of Chagas disease (93), also binds C1 and inhibits the CP. Most likely TiCalr in saliva helps to control the activation of host C, present in the blood meal and consequent digestive tract tissue damage (93). Another example is the tick *Amblyomma americanum*, which secretes Calr (AaCalr) while feeding (94). AaCalr also binds C1, but this interaction does not inhibit C activation (94). In ticks, such as *Boophilus micropus*, Calr (BmCalr) is present in saliva, is immunogenic in tick-infested bovines (95) and, similar to *Haemaphysalis qinghaiensis* Calr (HqCalr), it is secreted in their host during blood sucking, promoting a humoral response (96).

## Other TcCALR Functions in the Host-Parasite Interplay

As reviewed elsewhere (2), in addition to their roles in C evasion and infectivity, TcCalr and its N-terminal domain are anti-angiogenic in several experimental set ups (97–99). The anti-tumor effect of *T. cruzi* infection has been fully reproduced by exogenously administrated rTcCalr (97) and reverted by polyclonal anti-rTcCalr F(ab')_2_ antibodies (51). Native endogenous TcCalr, in the context of the parasite, also has an anti-tumor effect on *T. cruzi* infection, since mice inoculated with TA3-MTXR tumor cells, infected with *T. cruzi* trypomastigotes and treated with anti-TcCalr antibodies neutralize the anti-tumor effect of the infection (51). Most recently, we have proposed that TcCalr binds to canine transmissible venereal tumor (CTVT) cells and to a canine mammary carcinoma cell line, improving the immunogenicity of both tumors. These cells can be engulfed by macrophages and dendritic cells co-cultured with rTcCalr, accelerating its maturation and activating T cells (100). Similar to its human counterpart, TcCalr promotes wound healing in rats (101); however, whether this property correlates with the known anti-complement capacity of the parasite chaperone, is unknown.

## Summary

Calr is a multifunctional chaperone, resident in the ER, where it controls Ca^+2^ homeostasis. However, Calr has important roles outside the cells, because it is also secreted (58). Calr is highly conserved among plants and mammals and some of its functions are significant in host-pathogen interactions (2). Thus, an important function of Calr in microorganisms is its capacity to bind C1, with consequent inhibition of the CP of C (4) and promotion of infectivity (1, 4). More recently, these two important effects have also been described for Calr from a variety of protozoan and metazoan parasites (2). C1 binding allows the parasite to evade the C system and to promote engulfment of the parasite by mimicking a strategy used by apoptotic cells (12). TcCalr also has important functions related with the inhibition of angiogenesis and tumor growth, as revised elsewhere (51, 97, 98). Progress in the knowledge of Calr functions in different parasitic infections may be useful in the design of new therapies and/or vaccines.

## Author Contributions

GR-T, VF, and AF contributed equally to the generation of this review. GR-T prepared the figure. GR-T, VF, and AF edited the text. All authors contributed substantially to the writing and with published previous research included herein. All authors contributed to the article and approved the submitted version.

## Conflict of Interest

The authors declare that the research was conducted in the absence of any commercial or financial relationships that could be construed as a potential conflict of interest.
